# Probing the Roles of Calcium-Binding Sites during the Folding of Human Peptidylarginine Deiminase 4

**DOI:** 10.1038/s41598-017-02677-1

**Published:** 2017-05-25

**Authors:** Yi-Liang Liu, Chien-Yun Lee, Yu-Ni Huang, Hui-Yi Chen, Guang-Yaw Liu, Hui-Chih Hung

**Affiliations:** 1 Department of Life Sciences, National Chung Hsing University (NCHU), Taichung, Taiwan; 2 Institute of Biochemistry, Microbiology & Immunology, Chung Shan Medical University, and Division of Allergy, Immunology, and Rheumatology, Chung Shan Medical University Hospital, Taichung, Taiwan; 3 Graduate Institute of Biotechnology, National Chung Hsing University, Taichung, Taiwan; 4 Molecular and Biological Agricultural Sciences Program, Taiwan International Graduate Program, Academia Sinica, Taipei, Taiwan; 5 Biotechnology Center, NCHU, Taichung, Taiwan; 6 Institute of Genomics and Bioinformatics, NCHU, Taichung, Taiwan; 7 Agricultural Biotechnology Center, NCHU, Taichung, Taiwan

## Abstract

Our recent studies of peptidylarginine deiminase 4 (PAD4) demonstrate that its non-catalytic Ca^2+^-binding sites play a crucial role in the assembly of the correct geometry of the enzyme. Here, we examined the folding mechanism of PAD4 and the role of Ca^2+^ ions in the folding pathway. Multiple mutations were introduced into the calcium-binding sites, and these mutants were termed the Ca1_site, Ca2_site, Ca3_site, Ca4_site and Ca5_site mutants. Our data indicate that during the unfolding process, the PAD4 dimer first dissociates into monomers, and the monomers then undergo a three-state denaturation process via an intermediate state formation. In addition, Ca^2+^ ions assist in stabilizing the folding intermediate, particularly through binding to the Ca3_site and Ca4_site to ensure the correct and active conformation of PAD4. The binding of calcium ions to the Ca1_site and Ca2_site is directly involved in the catalytic action of the enzyme. Finally, this study proposes a model for the folding of PAD4. The nascent polypeptide chains of PAD4 are first folded into monomeric intermediate states, then continue to fold into monomers, and ultimately assemble into a functional and dimeric PAD4 enzyme, and cellular Ca^2+^ ions may be the critical factor governing the interchange.

## Introduction

The peptidylarginine deiminase (PAD; protein-arginine deiminase, EC 3.5.3.15) enzyme family catalyzes the Ca^2+^-dependent deimination of arginine to citrulline in proteins, concurrently producing ammonia^1, 2^. The citrullination catalyzed by this deiminase family is a type of post-translational modification^3–5^ that may have significant effects on the physiological functions of the target proteins and may play essential roles in cell differentiation^6^, nerve growth^7^, embryonic development^8^, cell apoptosis and gene regulation^9–13^.

PAD has various tissue distributions^14–19^. Five isoforms of PAD (PAD1-4 and PAD6) have been identified. PAD1 is found in the skin epidermis, where it citrullinates keratins and filaggrins^6, 20^. PAD2 is found in the brain, the nervous system and muscle tissues^15^. PAD3 is found in hair follicles, where it citrullinates trichohyalin for hair follicle hardening^16, 21^. PAD4 is found in granulocytes, monocytes and macrophages; it citrullinates histones H2A, H3 and H4 and nucleophosmin/B23^12, 13, 17, 22^. Finally, PAD6 is found in embryonic stem cells and oocytes^19^. PAD has broad substrate specificity. Filaggrin and histones H3 and H4 are the most extensively studied of the known PAD protein substrates^12, 13, 20, 23^. The citrullination sites of these proteins have been identified; thus, synthetic peptides derived from these proteins have been used to determine the sequence specificity of PAD protein substrates^24, 25^. The structures of PAD4 in a complex with various histone H3 and H4 peptides have been resolved, suggesting that PAD4 may recognize a structural motif on the protein surface rather than a specific consensus sequence^26^.

During the past ten years, studies of the PAD enzyme and citrullination have attracted much attention. First, high PAD4 activity and high levels of citrullinated proteins are highly related to the pathogenesis of an autoimmune disease known as rheumatoid arthritis (RA)^27^. An excess of autoantibodies against citrullinated proteins is often discovered in the blood of RA patients^28, 29^. A case control study by a Japanese group revealed that the *PAD4* haplotype that is associated with susceptibility to RA increases production of deiminated peptides that act as autoantigens^27, 30^. In particular, PAD4 is autocitrullinated *in vitro* and *in vivo*, and this modification inactivates its enzymatic function and enhances its recognition by human autoantibodies^31^. As a result, antibodies against these proteins can be used as diagnostic markers for RA. Furthermore, PAD inhibitors are drug development targets. The Cl- and F-amidine inhibitors have been synthesized to effectively inhibit PAD activity^32–34^.

Second, PAD4 is involved in histone demethylimination (citrullination) and causes a decrease in gene expression. Therefore, PAD4 is considered a transcriptional co-repressor^35^. Indeed, PAD4 is involved in the repression of p53 target genes by its interactions with the C-terminus of p53 and has other regulatory effects on p53 target genes^36, 37^. Because PAD4 has histone methylarginine deiminase activity, the enzyme results in the negative regulation of downstream p53 target genes, including the p21 protein. PAD4 functions as a p53 corepressor; therefore, inhibitors of this enzyme are considered to be potential treatments for cancer. The synthetic Cl- and F-amidine inhibitors have also been examined for their inhibitory effects on PAD4 activity and used to evaluate cancer cell survival rates^32, 37–39^.

Multiple X-ray structures of PAD4 complexed with ligands and calcium ions have been solved and are available in the Protein Data Bank^26, 40^. PAD4 is a homodimeric enzyme with an elongated rubber boot structure. Each monomer contains a separate active site with two calcium ions, Ca1 and Ca2, in the C-terminal domain and a distinct binding region with three additional calcium ions, Ca3, Ca4 and Ca5, situated at the N-terminal domain. The calcium-free, calcium-bound and substrate-bound PAD4 structures indicate that the binding of Ca^2+^ ions to the acidic concave enzyme surface induces a conformational change that creates the active site cleft^40^. The sequence identities among the five PAD isoforms are approximately 50–55%, and the catalytic and calcium-binding residues are mostly conserved, suggesting that the isoforms may have similar tertiary structures and catalytic mechanisms.

Our recent studies of PAD4 demonstrate that the dimerization of the enzyme is essential for full enzymatic activity and calcium-binding cooperativity^41^. In addition, we also provided evidence that the binding of Ca^2+^ ions to the Ca3-, Ca4- and Ca5-binding sites plays a crucial role in achieving the correct geometry for full activation of the enzyme^42^. However, the exact role of these calcium-binding sites during the folding of PAD4 is not yet clear. To address these questions, multiple mutations were made at the calcium-binding sites, and these mutations were termed the Ca1_site, Ca2_site, Ca3_site, Ca4_site and Ca5_site mutants. Based on biophysical data from several types of measurements, we propose that an intermediate state is formed during the folding process of PAD4 and that this intermediate is mainly stabilized by the binding of Ca^2+^ ions to the N-terminal Ca^2+^-binding site.

## Results and Discussion

**Figure 1:**
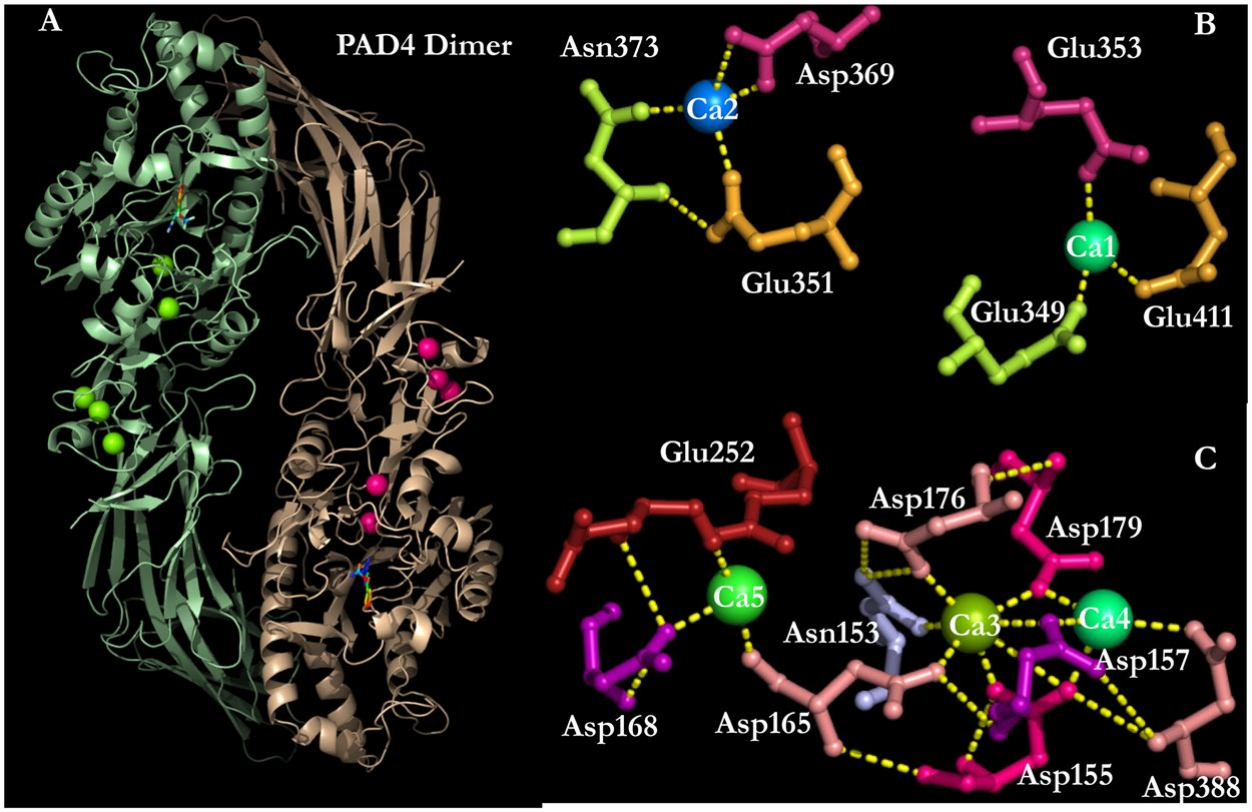
Five Ca^2+^-binding sites in the human PAD4 enzyme. Binding ligands for the five Ca^2+^-binding sites, which include the C-terminal calcium-binding sites, i.e., the Ca1_site and Ca2_site, and the N-terminal calcium-binding sites, i.e., the Ca3_site, Ca4_site and Ca5_site. This figure was generated using PyMOL^50^.

To explore the folding pathway of human dimeric PAD4 and to identify the specific roles of each of its calcium-binding sites (Ca1, Ca2, Ca3, Ca4 and Ca5; Fig. 1) during folding, PAD4 was subjected to urea-induced denaturation and renaturation, and multiple mutagenesis experiments were performed on its calcium-binding sites (Table 1).

**Table 1:** Mutations of the PAD4 enzyme at the Ca1-, Ca2-, Ca3-, Ca4- and Ca5-binding sites.

PAD4	Variants
Ca1_site mutant	Q349A/E353A/E411A
Ca2_site mutant	E351A/D369A/N373A
Ca3_site mutant	N153A/D155A/D157A/D165A/D176A/D179A
Ca4_site mutant	D155A/D157A/D179A/D388A
Ca5_site mutant	D168A/E252A

### Characteristics of human PAD4 and the Ca^2+^-binding-site mutant enzymes

The C-terminal Ca^2+^-binding sites, namely the Ca1_site and Ca2_site, are part of the active site of the enzyme (Fig. 1)^40^. The N-terminal Ca^2+^-binding sites, namely, the Ca3_site, Ca4_site and Ca5_site, are also required for enzymatic activity^42^. The kinetic parameters of the wild-type (WT) PAD4 and the calcium-binding-site mutant enzymes are presented in Table 2. The Michaelis constant of the *in vitro* substrate BAEE (*K*_m,BAEE_), the catalytic constant (*k*_cat_) and the specificity constant (*k*_cat_/*K*_m,BAEE_) of the WT enzyme were 0.57 mM, 14.5 s^−1^ and 25.4 mM^−1^ s^−1^, respectively. The binding of Ca^2+^ ions to the enzyme was cooperative with sigmoidal kinetics^38^. The half-saturation constant for Ca^2+^ (*K*_0.5,Ca_) and the degree of cooperativity of calcium binding (*h*) for the WT enzyme were 0.28 mM and 1.7, respectively.

**Table 2:** Kinetic parameters of PAD4 and the calcium-binding-site mutant enzymes.

PAD4	*K*_m,BAEE_^a^ (mM)	*k*_cat_^a^ (s^−1^)	*k*_cat_/*K*_m,BAEE_ (s^−1^mM^−1^)	*K*_0.5,Ca_ (mM)	*h* coefficient
WT	0.57 ± 0.33	14.5 ± 0.06	25.4	0.28 ± 0.05	1.7 ± 0.2
Ca1_site mutant	1.22 ± 0.40	0.02 ± 0.44	0.02	0.58 ± 0.27	1.0 ± 0.5
Ca2_site mutant	1.30 ± 0.15	1.45 ± 0.02	1.12	1.23 ± 0.44	1.0 ± 0.2
Ca3_site mutant	3.28 ± 0.81	1.84 ± 0.01	0.56	1.20 ± 0.37	1.0 ± 0.2
Ca4_site mutant	6.52 ± 2.40	0.07 ± 0.01	0.01	2.21 ± 0.38	1.0 ± 0.2
Ca5_site mutant	1.49 ± 0.08	4.54 ± 0.07	3.05	3.25 ± 0.09	1.8 ± 0.1

All the calcium-binding-site mutant enzymes displayed poor catalytic efficiency (Table 2), which is not surprising for the Ca1_site and Ca2_site mutants because Ca^2+^ ions were bound to these sites for catalysis. The *K*_m,BAEE_, *k*_cat_ and *k*_cat_/*K*_m,BAEE_ values of the Ca1_site mutant were 1.22 mM, 0.02 s^−1^ and 0.02 mM^−1^ s^−1^, respectively, and these values for the Ca2_site mutant were 1.3 mM, 1.45 s^−1^ and 1.12 mM^−1^ s^−1^, respectively (Table 2). Compared with that of the WT, the *K*_m,BAEE_ values of the Ca1_site and Ca2_site mutants increased approximately 2-fold, but the *k*_cat_/*K*_m,BAEE_ values of these two mutants severely decreased (Table 2), clearly showing the catalytic role of these two calcium ions. This role is especially obvious for the Ca1_site mutant. The overall catalytic efficiency of this mutant was just 0.1% of the WT efficiency (Table 2). The cooperative binding of calcium ions to the Ca1_site and Ca2_site mutants was completely abolished with an *h* value of 1.0.

For the Ca3_site, Ca4_site and Ca5_site mutants, the *K*_m,BAEE_ values were elevated with significantly decreased *k*_cat_ and *k*_cat_/*K*_m,BAEE_ values. For the Ca3_site mutant, the *K*_m,BAEE_, *k*_cat_ and *k*_cat_/*K*_m,BAEE_ values were 3.28 mM, 1.84 s^−1^ and 0.56 mM^−1^ s^−1^, respectively; for the Ca4_site mutant, these values were 6.52 mM, 0.07 s^−1^ and 0.01 mM^−1^ s^−1^, respectively, and for the Ca5_site mutant, they were 1.49 mM, 4.57 s^−1^ and 3.05 mM^−1^ s^−1^, respectively (Table 2). The cooperative binding of calcium ions to the Ca3_site and Ca4_site mutants was also abolished with an *h* value of 1.0. The Ca5_site mutant, however, is the only mutant that retained a slightly level of catalytic activity and cooperativity with an *h* value of 1.8, similar to that of the WT.

Although Ca3_site, Ca4_site and Ca5_site are not thought to be catalytic sites, mutations abolishing these binding sites severely affected the enzyme catalysis and increased the *K*_m,BAEE_ values by more than 6-fold (Table 2), indicating that the binding of calcium ions to these N-terminal Ca^2+^-binding sites may have profound effects on the precise conformation of the catalytic site. This effect is noticeable for the Ca4_site mutant, for which the *K*_m,BAEE_ value increased more than 10-fold over that of the WT (Table 2). Based on these findings, the Ca3_site and Ca4_site are crucial for the catalytic function of the enzyme by binding Ca^2+^ to ensure the correct conformation of the active site.

The PAD4 WT exhibited a stable dimer with a dissociation constant between monomer and dimer (*K*_d_) of 0.25 μM, and these calcium-binding-site mutants were also present as dimers with *K*_d_ values similar to that of the WT enzyme (Fig. S1 and Table S1). Although mutations in these calcium-binding sites did not affect the dimerization of PAD4, the calcium-binding-site mutants exhibited inactive dimers (Table 2), suggesting that the folding pathway of these mutants may be different from that of the WT enzyme.

### Unfolding and refolding of the WT PAD4 enzyme

**Figure 2:**
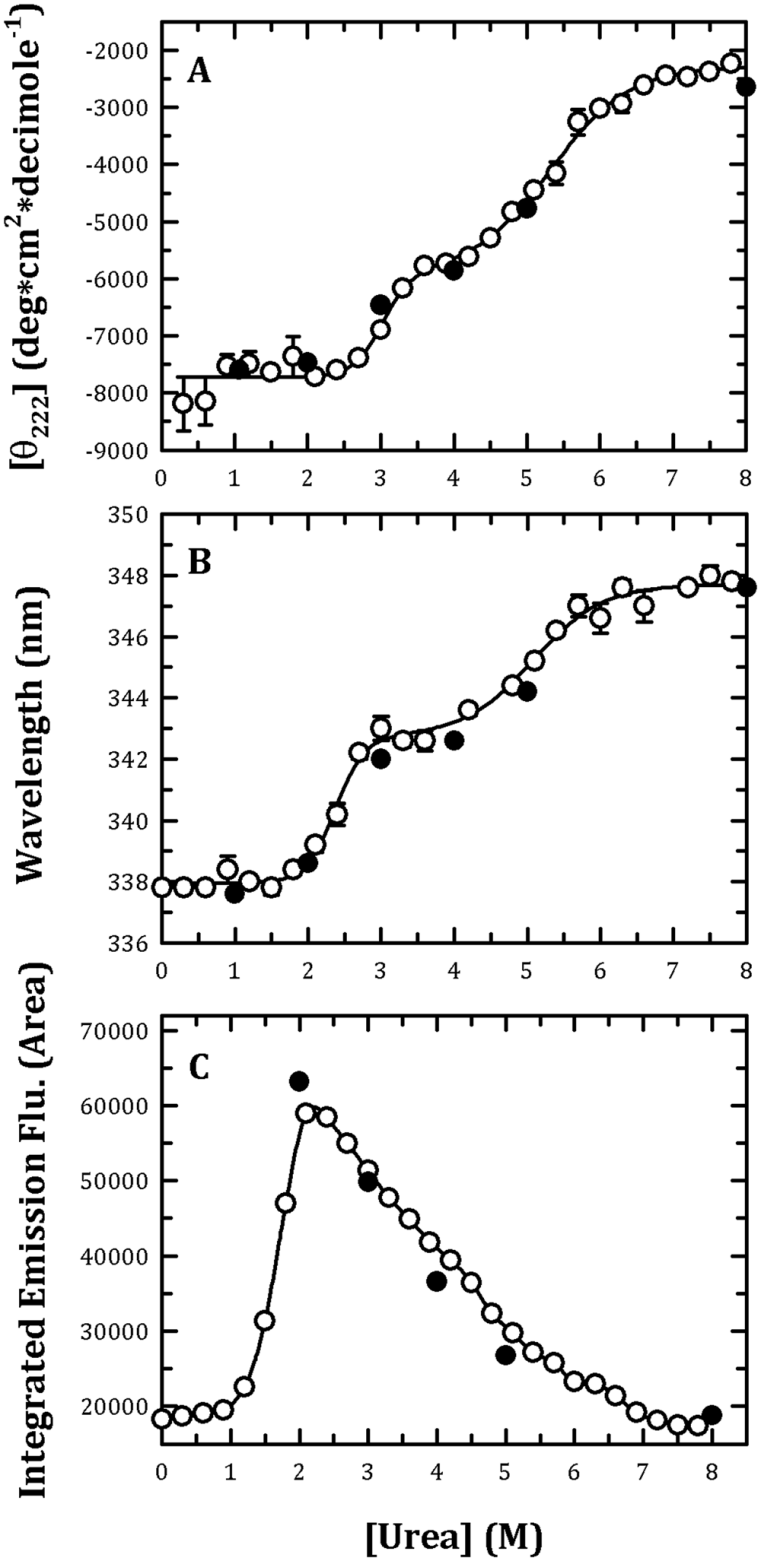
Monitoring of the urea-induced unfolding and refolding of human PAD4 WT enzyme by CD spectrometry and the intrinsic protein fluorescence. The PAD4 WT enzyme in the presence of 10 mM Ca^2+^ was treated with various concentrations of urea in 50 mM Tris-HCl buffer (pH 7.4) at 25 °C for 16 h and then monitored through CD spectrometry (**A**), fluorescence (**B**) or ANS fluorescence (**C**). Open circles: the PAD4 enzyme was denatured with different concentrations of urea. Closed circles: the PAD4 enzyme was completely denatured with 8 M urea and then renatured by diluting the urea concentration to 5, 4, 3, 2 and 1 M, as indicated in the figures. The experimental data in (**A**,**B**) were fitted by either a two-state or three-state model. The fit results and residues are shown as a solid line with error bars.

To investigate the folding pathway of PAD4, the urea-induced denaturation and renaturation of the WT enzyme was first examined. The conformational changes of the PAD4 enzyme were monitored by CD and intrinsic fluorescence (Fig. 2), and the thermodynamic parameters are shown in Table 3. The unfolding curve measured from the far-UV CD signals (molar ellipticity at 222 nm) was biphasic (Fig. 2A, open circles), and the urea concentrations at half-maximal denaturation, [Urea]_0.5_, for the first and second phases ([Urea]_0.5,N→I_ and [Urea]_0.5,I→U_, respectively) were approximately 3.0 and 5.3 M, respectively (Table 3). The intermediate state was achieved with approximately 3.5–4.0 M urea (Fig. 2A). Similar results were observed in the fluorescence experiments. The unfolding curve measured from the average fluorescence emission wavelengths was biphasic (Fig. 2B, open circles), with [Urea]_0.5_ values of 2.4 M for the first phase and 6.0 M for the second phase (Table 3). The intermediate state appeared in the presence of approximately 3–4 M urea (Fig. 2B).

**Table 3:** Thermodynamic parameters derived from the circular dichroism **(**CD**)** and fluorescence measurements of PAD4 and the calcium**-**binding**-**site mutant enzymes.

	*Midpoint of Urea*-*induced Denaturation*					
	CD measurement			Fluorescence measurement		
PAD4	[Urea]_0.5,N→I_ (M)	[Urea]_0.5,I→U_ (M)	[Urea]_0.5,N→U_ (M)	[Urea]_0.5,N→I_ (M)	[Urea]_0.5,I→U_ (M)	[Urea]_0.5,N→U_ (M)
WT	3.0 ± 1.6	5.3 ± 1.5	—	2.4 ± 0.8	6.0 ± 2.8	—
Ca1_site mutant	2.6 ± 1.0	5.7 ± 2.1	—	2.0 ± 1.5	5.3 ± 2.7	—
Ca2_site mutant	2.9 ± 1.0	6.3 ± 3.4	—	1.3 ± 1.3	4.1 ± 1.0	—
Ca3_site mutant	—	—	4.7 ± 1.1	—	—	4.1 ± 0.9
Ca4_site mutant	—	—	5.1 ± 1.4	—	—	4.9 ± 2.5
Ca5_site mutant	1.6 ± 1.2	5.3 ± 1.2	—	1.1 ± 0.8	4.6 ± 1.9	—

These values were derived from the circular dichroism data presented in Fig. 5A–E.

These values were derived from the fluorescence data presented in Fig. 5F–J.

The PAD4 enzyme was completely denatured with 8 M urea and then renatured by diluting the urea concentration to 5, 4, 3, 2 and 1 M (Fig. 2, closed circles). The refolding CD spectra for PAD4 WT enzyme in the native (0 M urea, black line), partially unfolded (1 M urea, red line), unfolded (8 M urea, blue line) and refolded (denatured with 8 M urea then renatured with 1 M urea, green line) states are shown in Fig. S2. Basically, the CD profile of the refolding curve (green line) was similar to the native (black line and partially unfolded states (red line). Thus, the value at 222 nm was used to present the unfolding/refolding status. As determined through monitoring the molar ellipticity at 222 nm or the fluorescence emission wavelength, the signals of the PAD4 enzyme reached the respective denatured condition (Fig. 2A,B, closed circles, respectively), indicating that the unfolding-refolding of PAD4 is a reversible process.

The ANS fluorescence of the urea-induced denaturation of PAD4 was also determined. ANS is generally employed as a sort of hydrophobic probe on protein surfaces. The changes in the ANS fluorescence for the PAD4-WT enzyme with increasing concentrations of urea were measured (Fig. 2C). A bell-shaped curve with a single peak that occurred at approximately 2 M urea and was followed by a shoulder was observed. The ANS fluorescence of PAD4 increased to a maximal value at approximately 2 M urea, indicating that some hydrophobic regions were largely exposed in the presence of 2 M urea, although no significant changes were detected using CD and fluorescence (Fig. 2A,B, respectively).

**Figure 3:**
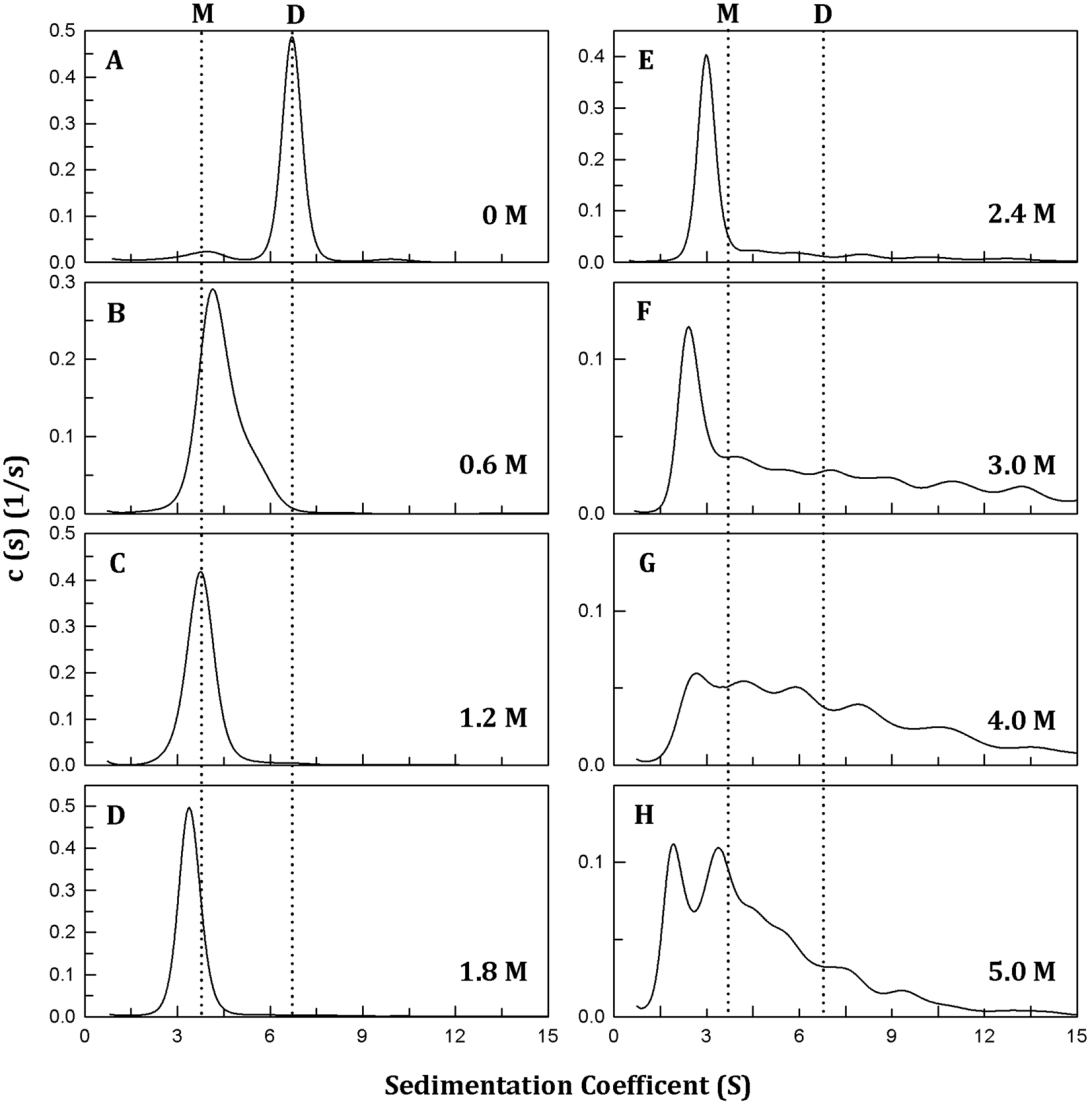
Continuous sedimentation coefficient distributions of human PAD4 WT enzyme during urea denaturation. The PAD4 WT enzyme (0.3 mg/ml) in the presence of 10 mM Ca^2+^ was treated with various concentrations of urea in 50 mM Tris-HCl buffer (pH 7.4) at 25 °C for 16 h: (**A**) 0 M urea, (**B**) 0.6 M urea, (**C**) 1.2 M urea, (**D**) 1.8 M urea, (**E**) 2.4 M urea, (**F**) 3.0 M urea, (**G**) 4.0 M urea and (**H**) 5.0 M urea.

**Figure 4:**
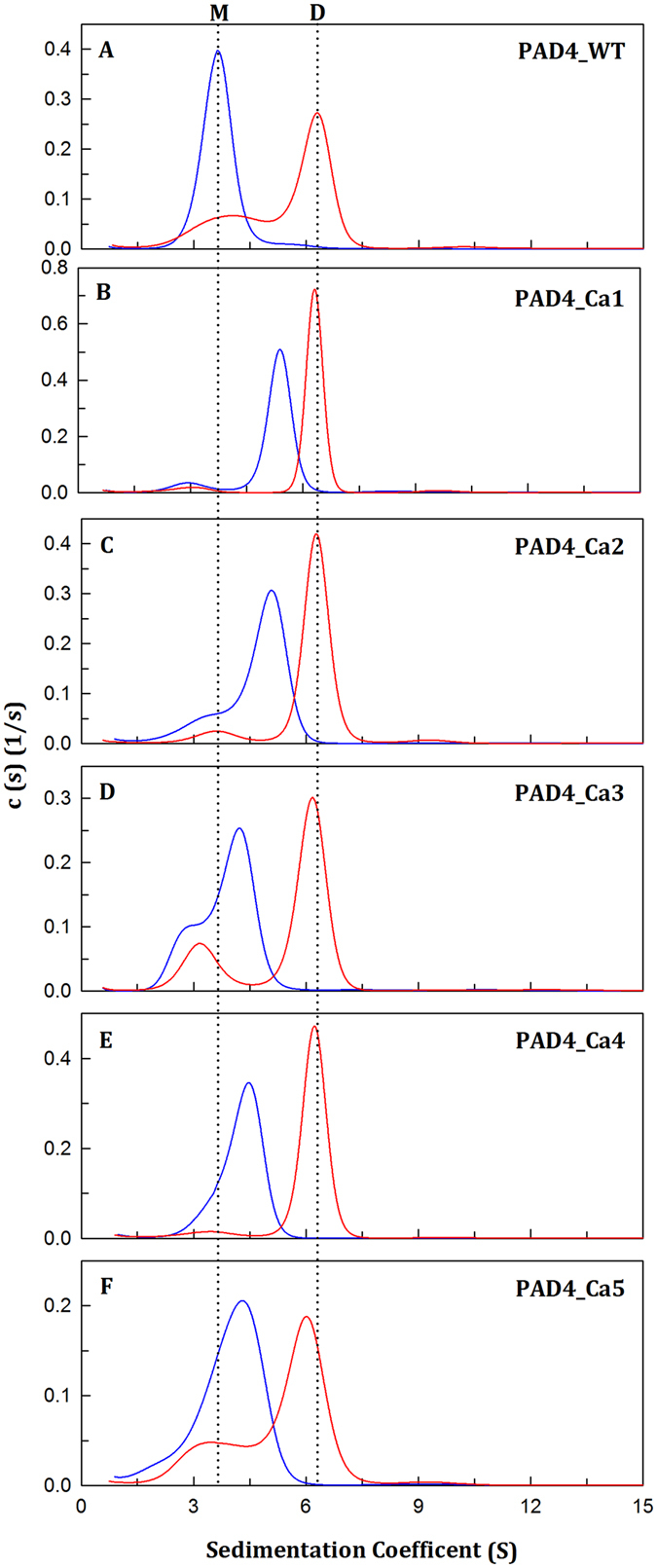
Dissociation-reassociation of human PAD4 WT and the calcium-binding-site mutant enzymes. The PAD4 WT and the calcium-binding-site mutant enzymes were treated with 1.2 M urea in 50 mM Tris-HCl buffer (pH 7.4) at 25 °C for 16 h, and the protein samples were then diluted 10-fold to reduce the urea concentration to 0.12 M. (**A**) PAD4_WT. (**B**) PAD4_Ca1. (**C**) PAD4_Ca2. (**D**) PAD4_Ca3. (**E**) PAD4_Ca4. (**F**) PAD4_Ca5. Blue line: the enzyme treated with 1.2 M urea. Red line: 10-fold dilution of the 1.2 M urea-treated enzyme (0.12 M urea).

We examined the quaternary structure changes of PAD4 during urea-induced denaturation by analytical ultracentrifugation (AUC). Figure 3 shows the continuous sedimentation coefficient distribution of PAD4 during urea denaturation. The sedimentation coefficients of 3.7 S and 6.8 S correspond to monomers and dimers, with molecular weights of 37 and 74 kDa, respectively. The quaternary structure of the WT enzyme displayed a dimer with a sedimentation coefficient of 6.8 S (Fig. 3A). However, the PAD4 dimer is sensitive to urea denaturation because the enzyme was significantly dissociated with 0.6 M urea (Fig. 3B). The enzyme completely dissociated into monomers with 1.2–1.8 M urea (Fig. 3C,D). At higher concentrations of urea, the size distribution plots of the enzyme became broad and irregular, indicating the unfolding of the monomeric enzyme (Fig. 3E–H). The 1.2 M urea-treated PAD4 enzyme, as previously mentioned, was found to exist as a monomer (Fig. 4A, blue line), and diluting the urea concentration from 1.2 M to 0.12 M caused the enzyme to re-associate into dimers (Fig. 4A, red line), indicating that the urea-induced dissociation of PAD4 is reversible. Furthermore, the dissociation process of PAD4 may correspond to the increased ANS fluorescence observed with 2 M urea (Fig. 2C) because the dimeric enzyme was completely dissociated into monomers in the presence of 2 M urea (Fig. 3), indicating that some hydrophobic regions in the dimer interface of PAD4 were exposed gradually during its dissociation from a dimer to monomers. In fact, a significant hydrophobic interface is present in the PAD4 dimer. Several hydrophobic amino acid residues are located at the dimer interface, including Y435, F541, W548 and F576 in the A subunit (colored in green; Fig. S3) and L6, L279, V283, V284 and F285 in the B subunit (colored in purple; Fig. S3). We have identified the hydrophobic amino acid residues at the dimer interface that significantly affected dimerization, particularly L6, L279 and V283^43^. Therefore, when the enzyme dissociated during urea-induced denaturation, the hydrophobic interface was gradually exposed, and then the hydrophobic probe ANS bound to this region, allowing us to monitor the dissociation of the enzyme.

Based on the denaturation data from the CD (Fig. 2A), fluorescence (Fig. 2B) and AUC measurements (Fig. 3), we suggest that the dissociation and denaturation processes of PAD4 are separate: the enzyme first dissociates from a dimer to a monomer at <2 M urea, and the monomer then begins to denature at higher urea concentrations (Fig. 2). The ANS fluorescence clearly increased to reach a maximal value at approximately 2 M urea; in the presence of this concentration of urea, the PAD4 dimer completely dissociated into monomers in the AUC experiments (Fig. 3), although dissociation was not detected in CD and fluorescence titration experiments (Fig. 2). This finding suggests that the first transition reflects the denaturation of the enzyme rather than the dissociation of the dimers and that dissociation of the dimers to monomers exposes the buried region in the dimer interface. Furthermore, the midpoints detected by CD and fluorescence, either between N and I or I and U, are close to each other (Table 3), suggesting that the enzyme truly follows a three-state unfolding process involving an intermediate that appeared at a urea concentration of 3–4 M (Fig. 2). In addition, the renaturation data from the CD (Fig. 2A, closed circles), fluorescence (Fig. 2B, closed circles) and AUC measurements (Fig. 4A) revealed that the urea-induced denaturation and dissociation of PAD4 is a reversible process; hence, the folding pathway of PAD4 can be characterized by tracing the unfolding of PAD4.

### Unfolding and refolding of the catalytic Ca^2+^-binding-site mutant enzymes (Ca1_site and Ca2_site mutants)

**Figure 5:**
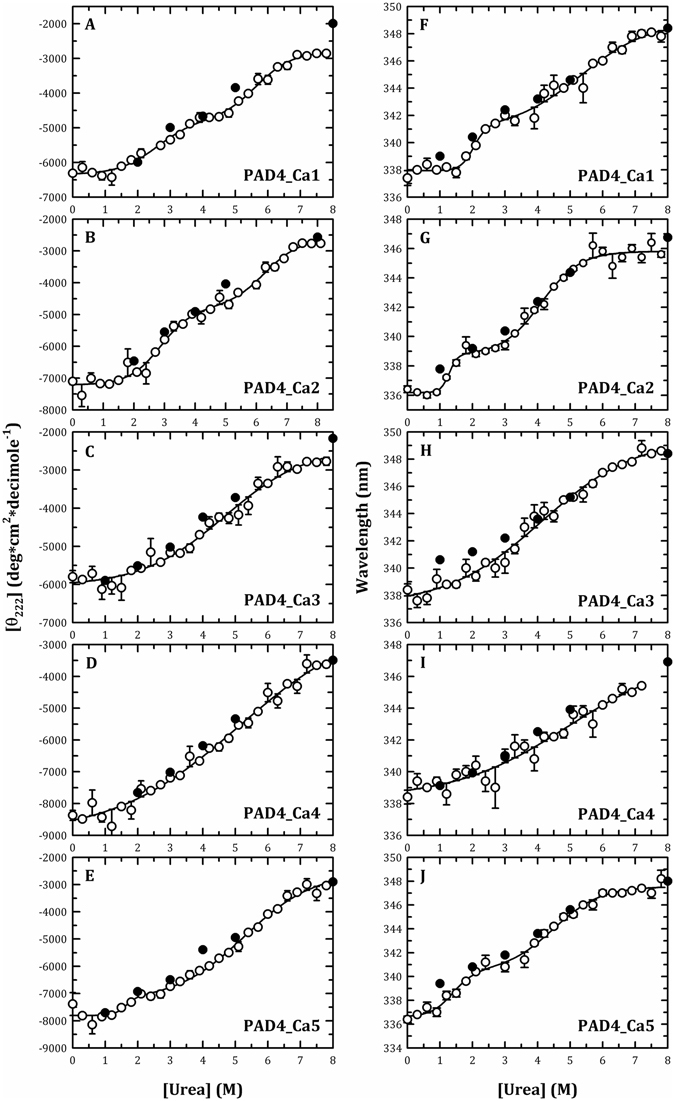
Monitoring of the urea-induced unfolding and refolding of the human PAD4 calcium-binding-site mutant enzymes by CD spectrometry and the intrinsic protein fluorescence. The PAD4 calcium-binding-site mutant enzymes in the presence of 10 mM Ca^2+^ were treated with various concentrations of urea in 50 mM Tris-HCl buffer (pH 7.4) at 25 °C for 16 h and then monitored through CD spectrometry (Panels (A–E)) or fluorescence (Panels (F–J)). Panels (A and F) PAD4_Ca1. Panels (B and G) PAD4_Ca2. Panels (C and H) PAD4_Ca3. Panels (D and I) PAD4_Ca4. Panels (E and J) PAD4_Ca5. Open circles: the PAD4 enzyme was denatured with different concentrations of urea. Closed circles: the PAD4 enzyme was completely denatured with 8 M urea and then renatured by diluting the urea concentration as indicated in the figures. All the data were fitted by either a two-state or three-state model. The fit results and residues are shown as a solid line with error bars.

To probe the role of the calcium-binding sites during the folding of PAD4, the urea-induced denaturation and renaturation of the calcium-binding-site mutants were examined. The conformational changes of the Ca1_site and Ca2_site mutants during urea-induced denaturation was monitored by far-UV CD (open circles in Fig. 5A,B, respectively) and fluorescence signals (open circles in Fig. 5F,G, respectively), and the thermodynamic parameters were determined (Table 3). The urea denaturation curves of the Ca1_site and Ca2_site mutants were also biphasic, similar to that of the WT enzyme (Fig. 2, open circles). The monitoring of the unfolding process by far-UV CD signals revealed that [Urea]_0.5_ values of the Ca1_site and Ca2_site mutants were 2.6 and 2.9 M, respectively, for the first phase (3.0 M for WT) and 5.7 M and 6.3 M, respectively, for the second phase (5.3 M for WT; Table 3). The intermediate state of the Ca1_site and Ca2_site mutants appeared in the presence of approximately 4 M urea (3.5–4.0 M for WT). The monitoring of the unfolding process by fluorescence signals showed that the [Urea]_0.5_ values of the Ca1_site and Ca2_site mutants were 2.0 and 1.3 M, respectively, for the first phase (2.4 M for WT) and 5.3 M and 4.1 M, respectively, for the second phase (6.0 M for WT; Table 3). The intermediate state of the Ca1_site and Ca2_site mutants appeared in the presence of approximately 2.5–3 M urea (3.0–4.0 M for WT). In summary, the unfolding patterns of the Ca1_site and Ca2_site mutants are similar to those of the WT protein, which followed a three-state unfolding pathway, although the intermediate states of these two mutants appeared earlier than the WT protein, as determined using fluorescence monitoring.

The Ca1_site and Ca2_site mutants were refolded through a reverse of unfolding path. After denaturation with 8 M urea, the mutant enzymes could be renatured by diluting the urea concentrations. The signals for protein renaturation, monitored through either the molar ellipticity at 222 nm (closed circles in Fig. 5A,B, respectively) or the fluorescence emission wavelength (closed circles in Fig. 5F,G, respectively), returned to values similar to those obtained for the respective denatured condition, indicating that these two mutants could refold to a native state similar to the WT enzyme. The 1.2 M urea-treated Ca1_site and Ca2_site mutants were dissociated (Fig. 4B,C, blue lines, respectively), and diluting the urea concentration from 1.2 M to 0.12 M caused the enzyme to re-associate into dimers (Fig. 4B,C, red lines, respectively), similar to the pattern found for the WT enzyme (Fig. 4A, red line). According to the abovementioned data, the folding of the tertiary structures and the assembly of the quaternary structures of the Ca1_site and Ca2_site mutants are similar to those of the WT protein, which follows a three-state pathway with an intermediate. The destruction of the catalytic Ca^2+^-binding sites of PAD4 or the diminished binding of Ca^2+^ ions to the Ca1_site and Ca2_site severely affected enzyme catalysis but not the formation of the intermediate, which is critical for the correct folding of PAD4. Therefore, the binding of calcium ions to the Ca1 and Ca2 sites mainly contribute to catalysis and are less important for structural stability.

### Unfolding and refolding of the non-catalytic Ca^2+^-binding-site mutant enzymes (Ca3_site, Ca4_site and Ca5_site mutants)

In contrast to the WT protein and the Ca1_site and Ca2_site mutants, the unfolding pattern of the Ca3_site and Ca4_site mutants displayed a monophasic denaturation curve with a broad shoulder, implying that an unstable intermediate may exist in equilibrium. The monitoring of the unfolding process by far-UV CD signals (open circles in Fig. 5C,D, respectively) revealed that the [Urea]_0.5_ values for the Ca3_site and Ca4_site mutants were 4.7 M and 5.1 M, respectively, and the monitoring of the unfolding process by fluorescence signals (open circles in Fig. 5H,I, respectively) showed [Urea]_0.5_ values for the Ca3_site and Ca4_site mutants of 4.1 and 4.9 M, respectively (Table 3).

The unfolding process of the Ca3_site and Ca4_site mutant enzymes was reversible. Most protein signals of renaturation monitored by CD (closed circles in Fig. 5C,D, respectively) or fluorescence (closed circles in Fig. 5H,I, respectively) returned to the values of the respective denatured condition. The Ca3_site and Ca4_site mutant enzymes in the presence of 1.2 M urea were dissociated into monomers (Fig. 4D,E, blue lines, respectively), and diluting the urea concentration from 1.2 M to 0.12 M caused the enzyme to re-associate from monomers into dimers (Fig. 4D,E, red lines, respectively), similar to the results found for the WT enzyme (Fig. 4A, red line). Based on these aforementioned data, the folding of the tertiary structures of the Ca3_site and Ca4_site mutants follows a two-state pathway with the formation of an unstable intermediate, and the assembly of the quaternary structures of these two mutants is similar to the WT protein. The destruction of the non-catalytic Ca^2+^-binding sites in PAD4 or the diminished binding of Ca^2+^ ions to the Ca3_site and Ca4_site severely affect the stability of the intermediate, which is critical for the correct folding of PAD4. Therefore, calcium binding to the Ca3 and Ca4 sites is crucial for tertiary structure folding.

The Ca5_site mutant displayed a biphasic denaturation curve. The monitoring of the unfolding process by far-UV CD signals (open circles in Fig. 5E) revealed [Urea]_0.5_ values for the Ca5_site mutant of 1.6 M for the first phase (3.0 M for WT) and 5.3 M for the second phase (5.3 M for WT; Table 3). The monitoring of the unfolding process by fluorescence signals (open circles in Fig. 5J) showed that the [Urea]_0.5_ values of the Ca5_site mutant was 1.1 M for the first phase (2.4 M for WT) and 4.6 M for the second phase (6.0 M for WT; Table 3). Although the unfolding pattern of the Ca5_site mutants was similar to that of the WT, the intermediate states were observed earlier than with the WT. Similar to the Ca1_site and Ca2_site of PAD4, the destruction of the Ca5_site did not severely influence the formation of an intermediate during the folding process.

The unfolding of the Ca5_site mutant enzyme is a reversible process. The signals of protein renaturation monitored by the molar ellipticity at 222 nm and the fluorescence emission wavelength returned to values similar to those of the respective denatured condition, indicating that the Ca5_site mutant could refold to a native state similar to the WT enzyme (closed circles in Fig. 5E,J, respectively). The Ca5_site mutant enzyme treated with 1.2 M urea was dissociated into monomers (Fig. 4F, blue line), and diluting the urea concentration from 1.2 M to 0.12 M caused the enzyme to re-associate into dimers (Fig. 4F, red line), similar to the pattern observed for the WT enzyme (Fig. 4A, red line). Thus, the folding of the tertiary structure and the assembly of the quaternary structure of the Ca5_site mutant are similar to the WT protein, suggesting that the binding of calcium to the Ca5_site is less important for the folding of the PAD4 protein.

### The folding pathway of the human PAD4 enzyme

**Figure 6:**
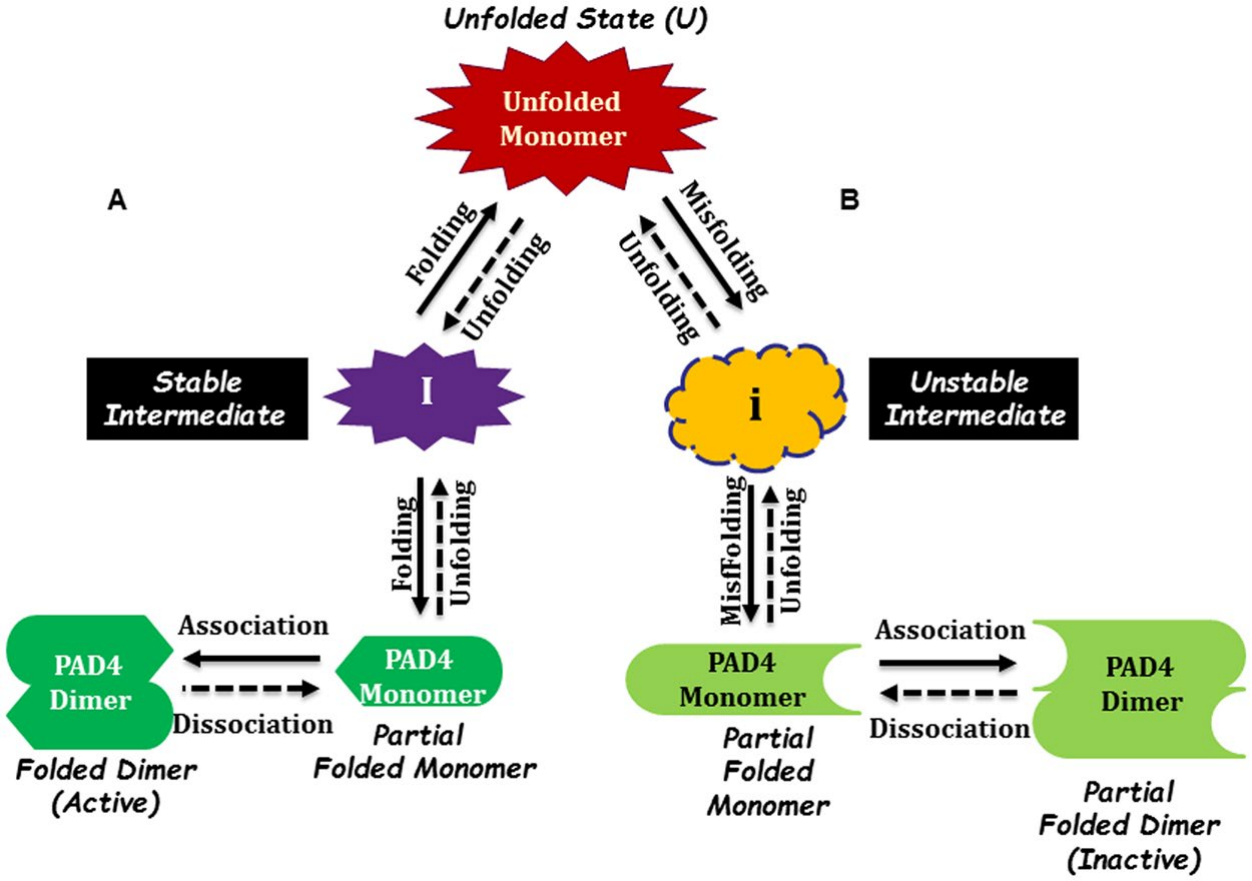
Proposed model of the PAD4 folding pathway. (**A**) Three-state folding pathway with the formation of a stable intermediate and assembly of dimers of the PAD4 WT, PAD4_Ca1, PAD4_Ca2 and PAD4_Ca5 mutant enzymes. (**B**) Two-state folding pathway with the formation of an unstable intermediate and assembly of dimers of PAD4_Ca3 and PAD4_Ca4 mutant enzymes.

Ca^2+^ ions are essential for the enzymatic activity and structural stability of the human PAD4 enzyme^40, 42^. Structural evidence suggests that the binding of Ca^2+^ to the enzyme induces significant conformational changes that generate the active enzyme^40^. The C-terminal Ca^2+^-binding sites, namely, the Ca1_site and Ca2_site, are believed to form part of the catalytic calcium-binding site of the enzyme^40^. Furthermore, the N-terminal Ca^2+^-binding sites, specifically the Ca3_site, Ca4_site and Ca5_site, have been shown to be required for enzymatic activity because they stabilize the conformational geometry of the enzyme^42^. This analysis of the unfolding-refolding process of PAD4 led us to propose a folding process for an active and dimeric PAD4 enzyme (Fig. 6).

During the unfolding process, the PAD4 dimer first dissociates into monomers, and the monomers subsequently follow a three-state denaturation process via the formation of an intermediate state. In addition, the binding of calcium ions to the Ca3_site and Ca4_site primarily affects the formation of a stable intermediate. Calcium ions are important for the folding of PAD4; in particular, the binding of calcium ions to the Ca3_ and Ca4_sites stabilizes the folding intermediate to ensure the correct folding of PAD4. The binding of calcium ions to the Ca1_site and Ca2_site may directly affect enzymatic catalysis and may be partially involved in achieving the correct active site conformation. Therefore, we propose a potential model for the folding pathway of PAD4 (Fig. 6). When a nascent polypeptide chain of PAD4 is synthesized, the unfolded PAD4 monomer is first folded into a monomeric intermediate state. The intermediate state continues to fold into a monomer. Two monomers then assemble into a dimeric PAD4 enzyme, which is fully functional for enzyme catalysis (Fig. 6, left). The binding of calcium ions to Ca3_site and Ca4_site is important for the formation of the intermediate state, which plays a crucial role during the folding of PAD4. If the Ca3_site or Ca4_site is mutated, the enzyme cannot fold properly, leading to an inactive enzyme (Fig. 6, right). The binding of calcium ions to the Ca1_site and Ca2_site is crucial for enzyme catalysis, and mutations that prevent these two sites from binding Ca^2+^ ions truly abolished the catalytic activity of PAD4 but did not severely affect the formation of an intermediate during the folding process (Fig. 6, left).

### The role of calcium ions in the folding pathway of PAD4

It appears much of the PAD4 enzyme is folded either in the absence or presence of Ca^2+^. Actually, the urea-induced denaturation of PAD4 holo-form (with 10 mM CaCl_2_) and apo-form (without 10 mM CaCl_2_) was examined through intrinsic fluorescence experiments, revealing that the two forms exhibited dissimilar conformational stabilities (Fig. S4). Although the urea denaturation curves of the holo-form and apo-form of PAD4 are apparently biphasic (Fig. S4, open and closed circles, respectively), the thermodynamic parameters and the curve transitions are quite different. The midpoints of the holo-form and apo-form of PAD4 were 2.4 and 0.4 M, respectively, for the first phase and 6.0 M and 5.2 M, respectively, for the second phase. The first transition of the apo-form emerged much earlier, and the intermediate state of the apo-form was much less obvious than those of the holo-form of PAD4, revealing that the holo-form and apo-form of PAD4 represent different conformations. We also performed the titration experiments of Ca^2+^ ions into the PAD4 WT enzyme which were monitored by the CD and fluorescence (Fig. S5). There were just slight changes in CD and fluorescence experiments during Ca^2+^ titration. Although Ca^2+^ ions enhance the PAD4 enzyme to produce a stable and active fold, the Ca^2+^-induced conformational changes of PAD4 could not be traced by CD or fluorescence. The native PAD4 enzyme without Ca^2+^ ions has CD or fluorescence spectra similar to the PAD4 enzyme with saturated Ca^2+^ ions.

These data coincide with the structural analysis of PAD4 in its Ca^2+^-free and Ca^2+^-bound form^40^. PAD4 can fold even the Ca^2+^ ions are absent^40^. However, an r.m.s. deviation is 1.1 Å observed by superimposing the backbone Cα atoms of Ca^2+^-free and Ca^2+^-bound PAD4. A number of loop regions are missing in the Ca^2+^-free structure but are visible in the Ca^2+^-bound structure. Binding of Ca^2+^ to Ca1 and Ca2 induces marked conformational changes that generate the active site cleft in the C-terminal domain. In addition, Ca^2+^ binding shows a marked difference in conformation in the region between Asn158 and Val171, which is disordered in Ca^2+^-free PAD4 but becomes ordered to form an α1-helix when Ca^2+^ binds to Ca3, Ca4, Ca5 and two water molecules in Ca^2+^-bound PAD4. As described above, PAD4 folds in the absence of Ca^2+^ but the folded state of the apo-form is different from the holo-form. Based on our data and structural analysis, we suggest that Ca^2+^ binding assists in stabilizing the intermediate state and protein structure. The pattern of the unfolding curve of the Ca1_site and Ca2_site mutants is similar to that of the holo-form of PAD4, and the pattern of the unfolding curve of the Ca3_site and Ca4_site mutants is similar to that of the apo-form of PAD4 (Fig. 5).

Finally, we suggest that the protein folding of PAD4 would occur at the intracellular concentrations of calcium ions (100 nM) found within a typical cell, where an equilibrium between unfolded monomers, partially folded intermediates, folded monomers and folded dimers exist (Fig. 6, left), and the enzyme displays very low activity under such low calcium ion concentrations. However, 10- to 100-fold increases in the calcium ion concentration, which can be observed during various cellular functions, causes the PAD4 enzymes to become activated, a process that may be attributed to the folding of most partially folded intermediates into monomers and their subsequent assembly into fully active dimers.

## Materials and Methods

### Expression and purification of recombinant WT and mutant PAD4

Human PAD4 cDNA was cloned into a pQE30 vector with an N-terminal His tag for purifying the overexpressed PAD4 enzyme. This vector contained an ampicillin-resistant gene and was transfected into the JM109 strain of *E*. *coli*. The expression of the PAD4 enzyme was induced with 1.0 mM isopropyl-1-thio-β-D-galactoside (IPTG), and the cells were harvested after overnight-incubation at 25 °C. Ni-NTA sepharose gel (Sigma, St. Louis, MO, USA), which was used to purify the target protein, was equilibrated with binding buffer (5 mM imidazole in 500 mM NaCl, 2 mM *β*-mercaptoethanol and 30 mM Tris-HCl, pH 7.6). The lysate-Ni-NTA mixture was loaded onto a column and washed with a stepwise procedure (5 and 10 mM imidazole in 500 mM NaCl, 2 mM *β*-mercaptoethanol and 30 mM Tris-HCl, pH 7.6) to remove undesired proteins. Lastly, PAD4 enzymes were washed out with elution buffer (250 mM imidazole, 500 mM NaCl, 2 mM *β*-mercaptoethanol and 30 mM Tris-HCl, pH 7.6). The purified enzyme was then subjected to buffer exchange with dialysis buffer (500 mM NaCl, 2 mM *β*-mercaptoethanol, and 50 mM Tris-HCl, pH 7.4) and concentrated using a centrifugal filter device (Amicon Ultra-15, Millipore) with a molecular weight cutoff of 50 kDa. The purity of the enzymes was examined by SDS-PAGE, and the protein concentrations were determined using the Bradford method^44^.

### Site-directed mutagenesis

Site-directed mutagenesis was conducted using a QuikChange™ kit (Stratagene, La Jolla, CA USA). This mutagenesis method was accomplished using *Pfu* DNA polymerase, an enzyme with high fidelity for DNA replication. The specific primers for mutagenesis were 25- to 45-mer oligonucleotides that bind specifically to the template DNA. Multiple mutagenic primers were used to make the calcium-binding-site mutants. For the Ca1_site, Ca2_site and Ca5_site mutants, three sets of primers for each were used; six and four sets of primers were used for the Ca3_site and Ca4_site mutants, respectively. The synthetic oligonucleotides used as mutagenic primers were the following:

N153A 5′-GCCATCCTGCTGGTGGCTTGTGACAGAGACAATC-3′,

D155A 5′-CCTGCTGGTGAACTGTGCTAGAGACAATCTCG-3′,

D157A 5′-GGTGAACTGTGACAGAGCTAATCTCGAATCTTCTGCC-3′,

D165A 5′-GAATCTTCTGCCATGGCTTGCGAGGATGATG-3′,

D168A 5′-GCCATGGACTGCGAGGCTGATGAAGTGCTTGAC-3′,

D176A 5′-GTGCTTGACAGCGAAGCTCTGCAGGACATGTCG-3′,

D179A 5′-GACAGCGAAGACCTGCAGGCTATGTCGCTGATGACCC-3′,

E252A 5′-CATGGACTTCTACGTGGCTGCCCTCGCTTTCCCG-3′,

Q349A 5′-GGATGACCAGTGGATGGCTGATGAAATGGAGATCGGC-3′,

E351A 5′-CCAGTGGATGCAGGATGCTATGGAGATCGGCTACATCC-3′,

E353A 5′-TGCAGGATGAAATGGCTATCGGCTACATCCAAGCCCC-3′,

D369A 5′-GCCCGTGGTCTTCGCTTCTCCAAGGAACAGAGGC-3′,

N373A 5′-GGTCTTCGACTCTCCAAGGGCTAGAGGCCTGAAGGAG-3′,

D388A 5′-GAGTGATGGGTCCAGCTTTTGGCTATGTAAC-3′, and

E411A 5′-CCTTTGGGAACCTGGCTGTGAGCCCCCCAGTCACAGTC-3′.

The PCR used 16–18 temperature cycles, and the desired mutant plasmids that included staggered nicks were produced. After the PCR reactions, the products were treated with DpnI to digest the PAD4 WT templates, and the nicked DNA with the anticipated mutations was transformed into the XL-1 strain of *Escherichia coli*.

### Enzyme assay and kinetic data analysis

The continuous assay method for measuring PAD4 activity has been reported earlier^45^. The regular reaction mixture for the spectrophotometric assay of PAD4 contained 10 mM benzoyl-L-arginine ethyl ester (BAEE) as an artificial substrate, 10 mM CaCl_2_, 2.5 mM dithiothreitol (DTT), 8.5 mM α-ketoglutarate (α-KG), 0.22 mM NADH and 8.4 U of glutamate dehydrogenase (GDH) in 100 mM Tris-HCl (pH 7.6) in a 1-ml cuvette at 25 °C. The appropriate amount of PAD4 was added to the assay mixture to start the reaction. After the enzyme was added to the reaction, the decline in absorbance at 340 nm was monitored continuously using a Perkin-Elmer Lambda-25 spectrophotometer. An enzyme unit was defined as the amount of enzyme that catalyzes the consumption of 1 μmol of NADH per min. An extinction coefficient of 6.22 cm^−1^ mM^−1^ at 340 nm for NADH was used in calculations. The *K*_m_ value of the BAEE substrate was determined by varying the BAEE concentration while maintaining constant concentrations of other components. The experimental data were analyzed using Prism 4.0. The sigmoidal curves of the [Ca^2+^] versus the initial velocities were fitted to the Hill equation, and the data were further analyzed to calculate the *K*_0.5,Ca_ value (i.e., the calcium concentration at half-maximal velocity) and the Hill coefficient (*h*), which were employed to assess the degree of cooperativity.

1 $$v=\frac{{V}_{{\rm{\max }}}{[C{a}^{2+}]}^{h}}{{K}_{0.5,Ca}^{h}+{[C{a}^{2+}]}^{h}}$$

All data fitting was performed using Sigma Plot 10.0 (Jandel, San Rafael, CA, USA).

### Equilibrium denaturation and renaturation of the PAD4 enzyme

Urea was used as the chemical denaturant for the denaturation and renaturation of PAD4. The PAD4 enzymes were preincubated with various concentrations of urea in 50 mM Tris-HCl buffer (pH 7.4) for 16 h at 25 °C to enable the unfolding reactions to reach equilibrium. Before urea denaturation, the enzyme was incubated with 10 mM Ca^2+^ to prevent the precipitation of PAD4. The urea-induced denaturation of PAD4 was performed in the presence of 10 mM CaCl_2_. For the renaturation experiments, the enzyme was first denatured with 8 M urea and subsequently renatured by diluting the urea concentration. The final concentrations of PAD4 and CaCl_2_ in the denaturation and renaturation experiments were maintained equal for further measurement of the conformational changes of the enzyme by equilibrium denaturation and renaturation in the presence of urea.

### Monitoring the urea-induced denaturation and renaturation of PAD4 and the calcium-binding-site mutant enzymes using circular dichroism spectrometry and fluorescence spectroscopy

Two techniques, circular dichroism (CD) and fluorescence spectroscopy, were employed to monitor the unfolding and refolding process of the enzymes.

CD signals was measured using a Jasco J-815 spectropolarimeter with 0.1-cm quartz cuvettes and a 1-mm slit width. The ellipticity (222 nm) of all samples was recorded to analyze the protein conformational changes during the urea denaturation. The mean residue ellipticity ((Θ)) at 222 nm was calculated using the following equation:

2 $$({\rm{\Theta }})=\frac{{\rm{MRW}}\times {\theta }_{\lambda }}{10\times 1\times c}$$

where MRW is the mean residue weight, θ_λ_ is the measured ellipticity in degrees at wavelength λ, l is the cuvette pathlength (0.1 cm), and c is the protein concentration in g/ml.

Fluorescence spectroscopy was performed using a Hitachi F-4500 FL luminescence spectrometer at 25 °C. The excitation wavelength was set to 290 nm, and the fluorescence emission spectra were recorded by scanning the emission from 300 to 400 nm. All spectra were corrected for buffer absorption and the Raman spectrum of water. The average emission wavelength (〈*λ*〉) was analyzed using the average emission wavelength method^46^ and calculated according to the following equation:

3 $$\langle \lambda \rangle =\frac{\sum {F}_{i}{\lambda }_{i}}{\sum {F}_{i}}$$

in which *F*_*i*_ is the fluorescence intensity at a specific emission wavelength (*λ*_*i*_).

### Monitoring the surface hydrophobicity of PAD4 denaturation through ANS fluorescence

An extrinsic fluorescent probe, ANS (8-anilino-l-naphthalene sulfonate), is used to detect the surface hydrophobic properties of a protein molecule. Therefore, the change in surface hydrophobicity of the WT human PAD4 during urea-induced denaturation was further measured through ANS fluorescence. The enzyme was denatured using various concentrations of urea for 16 h at 25 °C. ANS was then added to the protein sample, and the ANS fluorescence of the protein was then measured using a Hitachi F-4500 FL luminescence spectrometer at 25 °C. All spectra were corrected for buffer absorption. The excitation wavelength was set to 370 nm to monitor the changes in the surface hydrophobicity of the enzyme during the urea-induced unfolding process. The ANS fluorescence spectra were scanned from 400 to 600 nm, and the area of ANS fluorescence was integrated from 425 to 575 nm.

### Analysis of the urea-induced denaturation curve of PAD4 and the calcium-binding-site mutant enzymes

The analysis of the unfolding curves of the enzymes was performed as described by Pace^47^, assuming a two-state or three-state unfolding mechanism. For a two-state model, the ∆*G*_(H2O)_ and *m* values were estimated by fitting the data to the following equation:

4 $${{\rm{y}}}_{{\rm{obs}}}=\frac{{{\rm{y}}}_{{\rm{N}}}+{{\rm{y}}}_{{\rm{U}}}\,\exp \,\{-({{\rm{\Delta }}{\rm{G}}}_{({\rm{H2O}})}-{\rm{m}}\,[{\rm{D}}])/{\rm{RT}}\}}{1+\exp \,\{-{({\rm{\Delta }}{\rm{G}}}_{({\rm{H2O}})}-{\rm{m}}\,[{\rm{D}}])/{\rm{RT}}\}}$$

where *y*_obs_ denotes the observed signal change, and *y*_N_ and *y*_U_ represent the signals of the folded and unfolded states, respectively. ∆*G*_(H2O)_ denotes the intrinsic free energy change in the absence of denaturant, and *m* represents the dependence of ∆*G* on the denaturant. [D] denotes the denaturant concentration, *T* is the absolute temperature in degrees Kelvin, and *R* is the gas constant.

The denaturation curve was also analyzed using a three-state model. The ∆*G*_(H2O)_ and *m* values at each step were estimated by fitting the overall data to the following equation:

5 $${{\rm{y}}}_{{\rm{obs}}}=\frac{{{\rm{y}}}_{{\rm{N}}}+{{\rm{y}}}_{{\rm{I}}}\,\exp \,\{\,-\,{({\rm{\Delta }}{\rm{G}}}_{({\rm{H2O}}),{\rm{N}}\to {\rm{I}}}-{{\rm{m}}}_{{\rm{N}}\to {\rm{I}}}\,[{\rm{D}}])/{\rm{RT}}\}+{{\rm{y}}}_{{\rm{U}}}\,\exp \,\{\,-\,{({\rm{\Delta }}{\rm{G}}}_{({\rm{H2O}}),{\rm{N}}\to {\rm{I}}}-{{\rm{m}}}_{{\rm{N}}\to {\rm{I}}}\,[{\rm{D}}])/\text{RT}\}\ast \exp \,\{\,-\,{({\rm{\Delta }}{\rm{G}}}_{({\rm{H2O}}),{\rm{I}}\to {\rm{U}}}-{{\rm{m}}}_{{\rm{I}}\to {\rm{U}}}\,[{\rm{D}}])/\text{RT}\}}{1+\exp \,\{\,-\,{({\rm{\Delta }}{\rm{G}}}_{({\rm{H2O}}),{\rm{N}}\to {\rm{I}}}-{{\rm{m}}}_{{\rm{N}}\to {\rm{I}}}\,[{\rm{D}}])/\text{RT}\}+\exp \,\{\,-\,{({\rm{\Delta }}{\rm{G}}}_{({\rm{H2O}}),{\rm{N}}\to {\rm{I}}}-{{\rm{m}}}_{{\rm{N}}\to {\rm{I}}}\,[{\rm{D}}])/\text{RT}\}\ast \exp \,\{\,-\,{({\rm{\Delta }}{\rm{G}}}_{({\rm{H2O}}),{\rm{I}}\to {\rm{U}}}-{{\rm{m}}}_{{\rm{I}}\to {\rm{U}}}\,[{\rm{D}}])/\text{RT}\}}$$

where *y*_I_ represent the signals of the intermediate states, ∆*G*_(H2O),N→I_ and ∆*G*_(H2O),I→U_ denote the intrinsic free energy change for the native to intermediate (N → I) and for the intermediate to denatured (I → U) processes, respectively, and *m*_N→I_ and *m*_I→U_ are the *m* values for the corresponding processes. The urea concentration for half-denaturation of the protein, [Urea]_0.5_, was estimated by dividing ∆*G* by *m* ^47^.

### Quaternary structure analysis of PAD4 and the calcium-binding-site mutant enzymes by analytical ultracentrifugation

To examine the quaternary structural changes of the dimeric PAD4 enzyme during urea denaturation, sedimentation velocity experiments were performed using a Beckman Optima XL-A analytical ultracentrifuge (Beckman Coulter, CA, USA). Before ultracentrifugation, the protein sample was preincubated with various concentrations of urea (0–5.4 M) in the presence of 10 mM CaCl_2_ at 25 °C for 16 h. The sample (380 μl) and buffer (400 μl) solutions were separately loaded into the double sector centerpiece and placed in a Beckman An-50 Ti rotor. The experiments were performed at 20 °C and at a rotor speed of 42,000 rpm. The protein samples were monitored by the UV absorbance at 280 nm in continuous mode with a time interval of 480 s and a step size of 0.002 cm. Multiple scans at different time points were fitted to a continuous size distribution model by the program SEDFIT^48, 49^. All size distributions were solved at a confidence level of p = 0.95, a best fitted average anhydrous frictional ratio (*f*/*f*_*0*_), and a resolution N of 250 sedimentation coefficients between 0.1 and 20.0 S.

## Electronic supplementary material

Supplemental Information
