# Supplementary material for: Probing the Roles of Calcium-Binding Sites during the Folding of Human Peptidylarginine Deiminase 4
Source: Sci Rep. 2017 May 25;7:2429. doi: 10.1038/s41598-017-02677-1 (PMC5445078; doi:10.1038/s41598-017-02677-1)
Supplement: Supplementary file 1 — Supplemental Information [file 41598_2017_2677_MOESM1_ESM.pdf]

Probing the Roles of Calcium-Binding Sites during the Folding of Human Peptidylarginine Deiminase 4

Yi-Liang Liu, Chien-Yun Lee, Yu-Ni Huang, Hui-Yi Chen, Guang-Yaw Liu and Hui-Chih Hung

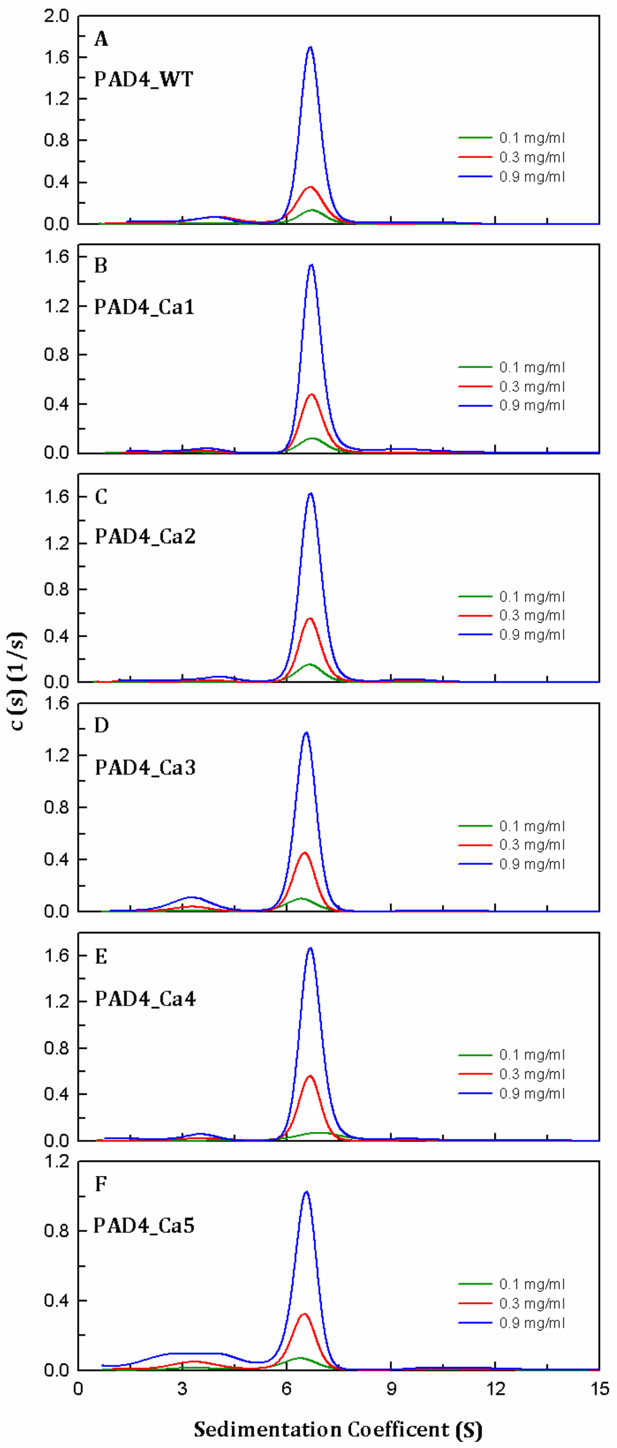

Figure S1. Continuous sedimentation coefficient distributions of PAD4 WT and the calcium-binding-site mutant enzymes.

(**A**) PAD4_WT. (**B**) PAD4_Ca1. (**C**) PAD4_Ca2. (**D**) PAD4_Ca3. (**E**) PAD4_Ca4. (**F**) PAD4_Ca5. Three protein concentrations (0.1, 0.3, and 0.9 mg/ml) were used, as indicated in the figure. The sedimentation velocity data were globally fit using the SEDPHAT program to obtain the dissociation constant (K_d) of the PAD4 dimer (Table S1).

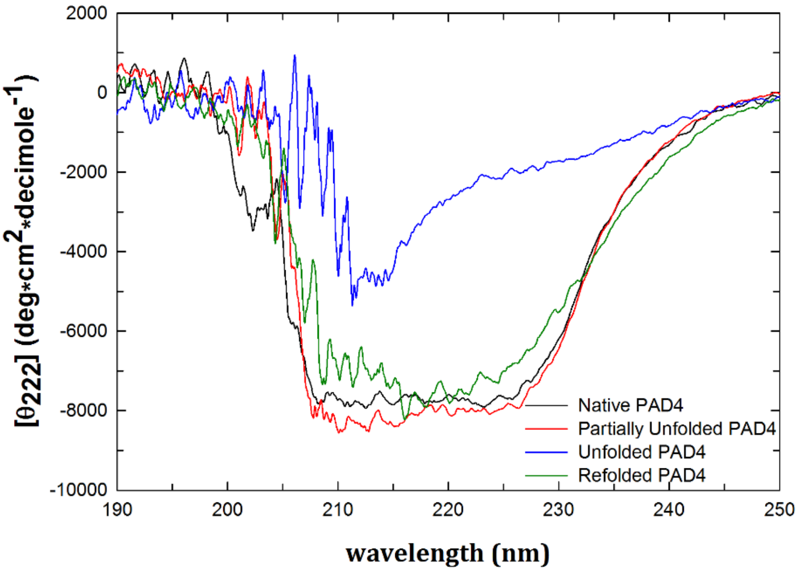

Figure S2. Full CD spectra of PAD4 WT enzyme under the denatured and renatured conditions.

The full CD spectra of the PAD4 enzyme in the native (0 M urea, black line), partially unfolded (1 M urea, red line), unfolded (8 M urea, blue line) and refolded (denatured with 8 M urea then renatured with 1 M urea, green line).

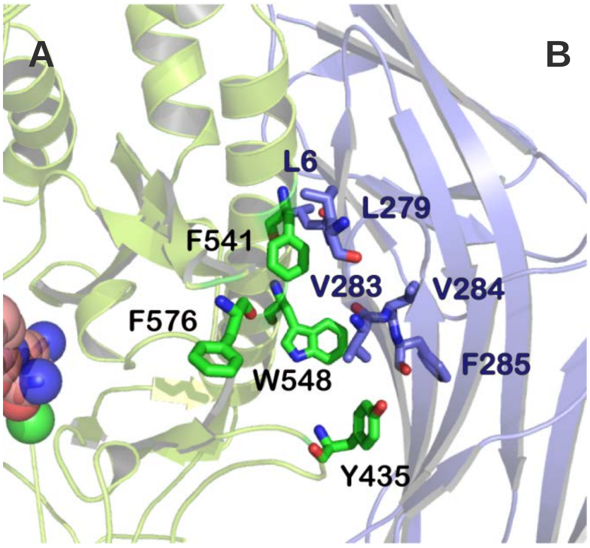

Figure S3. Hydrophobic interface in the PAD4 dimer.

Several hydrophobic amino acid residues in the dimer interface, including Y435, F541, W548 and F576 in the A subunit (colored in green) and L6, L279, V283, V284 and F285 in the B subunit (colored in purple).

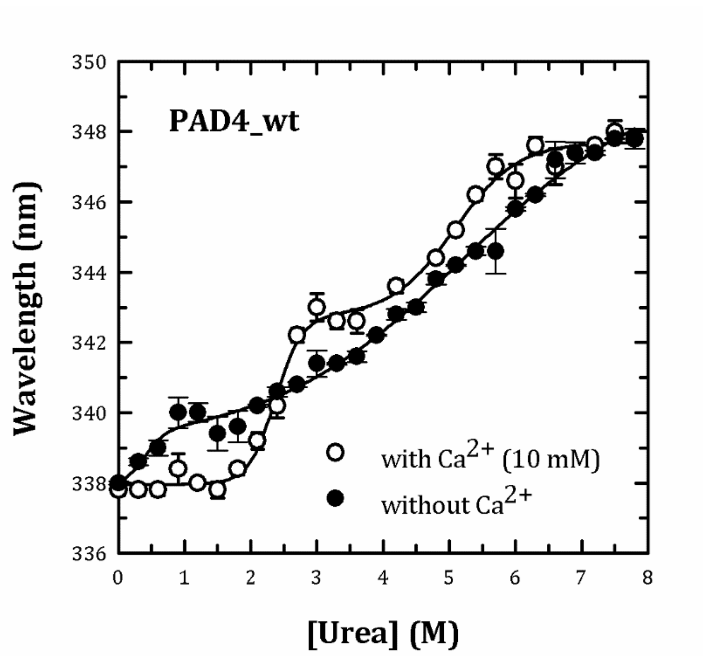

Figure S4. Monitoring of urea-induced denaturation of the holo-form and apo-form of PAD4 through intrinsic protein fluorescence.

PAD4 with 10 mM $CaCl_2$ (holo-form) or without 10 mM $CaCl_2$ (apo-form) was treated with various concentrations of urea in 50 mM Tris-HCl buffer (pH 7.4) at 25°C for 16 h and then monitored through intrinsic protein fluorescence measurements. Open circles: the PAD4 enzyme with 10 mM $CaCl_2$. Closed circles: the PAD4 enzyme without 10 mM $CaCl_2$. The experimental data were fitted by a three-state model. The fit results and residues are shown as a solid line with error bars.

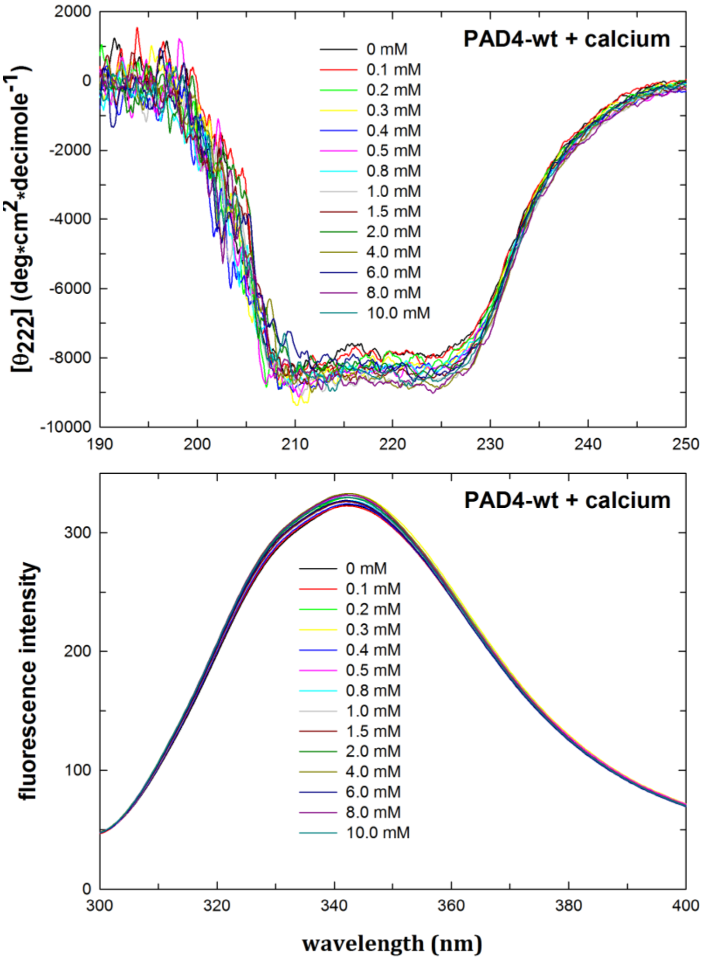

Figure S5. Monitoring of the titration of Ca^{2+} ions for PAD4 WT enzyme through CD and fluorescence.

PAD4 with various concentrations of $CaCl_2$ in 50 mM Tris-HCl buffer (pH 7.4) at 25°C for 16 h and then monitored through CD and intrinsic protein fluorescence measurements.

Table S1

Dissociation constant of the Ca1-, Ca2-, Ca3-, Ca4- and Ca5-binding site mutants

PAD4	K_d (μM)
Ca1_site mutant	0.16±0.001
Ca2_site mutant	0.15±0.001
Ca3_site mutant	0.16±0.001
Ca4_site mutant	0.41±0.004
Ca5_site mutant	0.17±0.002
